# Supplementary material for: Telomere Length Is Correlated with Resting Metabolic Rate and Aerobic Capacity in Women: A Cross-Sectional Study
Source: Int J Mol Sci. 2022 Nov 1;23(21):13336. doi: 10.3390/ijms232113336 (PMC9654753; doi:10.3390/ijms232113336)
Supplement: Supplementary file 1 [file ijms-23-13336-s001.zip › ijms-1956440-supplementary.pdf]

Supplementary Table S1. Demographic data, anthropometry, body composition, and physiological characteristics of the participants.

	**All (*n* = 134) Mean ± SD/ Median (IQR)**	**NW (*n* = 68) Mean ± SD/ Median (IQR)**	**OB (*n* = 66) Mean ± SD/ Median (IQR)**	***p* value**
Age (year)	40.5 (29.0 – 50.0)	39.5 (29.5 – 49.5)	41.0 (29.0 – 52.0)	0.774
Education level (%)				0.014
< high school	51 (38.1)	19 (27.9)	32 (48.5)	
≥ high school	83 (61.9)	49 (72.1)	34 (52.5)	
Menstruation (%)				0.547
Menstruation	88 (65.7)	43 (63.2)	45 (68.2)	
Menopause	46 (34.3)	25 (36.8)	21 (31.8)	
Marital status (%)				0.119
Single	41 (30.6)	26 (38.2)	15 (22.7)	
Married	88 (65.7)	39 (57.4)	49 (74.2)	
Divorced	5 (3.7)	3 (4.4)	2 (3.0)	
BM (kg)	62.4 (50.8 – 77.2)	51.6 ± 5.6	78.1 ± 8.0	< 0.001
Ht (m)	1.6 ± 0.1	1.6 ± 0.1	1.6 ± 0.1	0.619
BMI (kg/m^2)	23.6 (21.1 – 32.1)	21.1 (20.0 – 22.5)	32.1 (30.6 – 33.9)	< 0.001
W (cm)	86.0 (74.0 – 99.0)	73.7 ± 6.3	98.5 ± 7.8	< 0.001
W/H	0.8 ± 0.1	0.8 ± 0.1	0.9 ± 0.1	< 0.001
BF (%)	35.0 (28.6 – 38.8)	28.9 ± 3.9	39.0 ± 2.1	< 0.001
FM (kg)	19.8 (15.3 – 30.4)	15.0 ±2.9	30.6 ±4.2	< 0.001
FFM (kg)	42.5 (36.0 – 46.9)	36.6 ± 4.0	47.6 ± 4.2	< 0.001
RHR (/min)	68.0 (62.0 – 74.0)	67.0 (62.0 – 22.5)	69.0 (62.8 – 76.0)	0.226
SBP (mmHg)	111.7 ± 11.7	108.2 ± 12.2	115.4 ± 9.9	< 0.001
DBP (mmHg)	70.0 (66.0 – 76.0)	69.0 (64.0 – 75.0)	70.5 (66.0 – 77.5)	0.154
FBG (mmol/L)	5.4 (4.5 – 6.3)	5.0 (4.3 – 6.1)	5.8 (5.0 – 6.3)	0.002
HOMA-IR	0.1 (0.0 – 1.2)	0.1 (0.0 – 0.7)	0.4 (0.0 – 0.2)	0.016
HbA1c (%)	5.4 ± 0.4	5.2 (5.0 – 5.4)	5.6 (5.2 – 5.8)	< 0.001
TC (mmol/L)	10.5 ± 1.7	10.6 ± 1.3	10.4 ± 2.0	0.472
HDL-c (mmol/L)	2.9 ± 0.9	3.2 ± 0.9	2.7 ± 0.7	0.002
LDL-c (mmol/L)	6.3 ± 1.6	6.3 ± 1.3	6.3 ± 1.9	0.973
TG (mmol/L)	5.3 (4.2 – 6.7)	4.7 (3.8 – 5.9)	5.9 (5.1 – 7.3)	< 0.001
Cr (mg/dL)	0.7 (0.7 - 0.8)	0.7 (0.6 - 1.0)	0.8 (0.5 - 1.0)	0.604
SGPT (mg/dL)	14.1 (10.2 – 19.1)	17.0 ± 21.2	18.4 ± 13.0	0.126
EI (kcal/day)	1,716.7 ± 522.2	1,547.1 ± 416.8	1,891.4 ± 563.8	< 0.001
EE (kcal/day)	1282.2 (1,090.5 – 1381.5)	1202.4 (1,000.3 – 1366.9)	1305.1 (1,190.8 – 1395.2)	0.032

Data are expressed as mean ± SD / median (interquartile range, IQR) based on normal distribution. Independent t-test or Mann-Whitney U test was used for the normally and not normally distributed variables. Demographic data are expressed as frequency (%) the *p*-value was used chi-square. The *p*-value compares between NW and OB group.

Abbreviation: NW, normal weight; OB, obesity; BM, body mass; Ht, height; BMI, body mass index; W, waist circumference; W/H, waist to hip circumference ratio; BF, body fat; FM, fat mass; FFM, fat-free mass; RHR, resting heart rate; SBP, systolic blood pressure; DBP, diastolic blood pressure; FBG, fasting blood glucose; HOMA-IR, Homeostatic Model Assessment for Insulin Resistance; HbA1c, hemoglobin A1c; TC, total cholesterol; HDL-c, high-density lipoprotein cholesterol; LDL-c, low-density lipoprotein cholesterol; TG, triacylglycerol; Cr, creatinine; SGPT, Serum glutamic pyruvic transaminase; EI, energy intake; EE, energy expenditure.

Supplementary Table S2. Anthropometry, Body composition, metabolism, aerobic capacity, oxidative stress, inflammation, and RTL by age ranges.

		**20-29-year-old (*n* = 17NW, 17OB)**	**30-39-year-old (*n* = 17NW, 15OB)**	**40-49-year-old (*n* = 17NW, 17OB)**	**50-59-year-old (*n* = 17NW, 17OB)**
Age (yr)	NW	25.2 ± 2.5	33.9 ± 3.2***	44.8 ± 3.1***&&&	53.8 ± 3.1***&&&$$$
	OB	23.6 ± 2.9	35.8 ± 2.5***	45.5 ± 2.6***&&&	54.7 ± 2.3***&&&$$$
BM (kg)	NW	52.8 ± 5.6	54.5 ± 5.7	48.2 ± 5.1&&	50.9 ± 4.3
	OB	79.3 ± 7.5###	83.1 ± 8.8###	74.2 ± 7.5&&###	76.5 ± 6.1&###
BMI (kg/m^2)	NW	21.1 (20.3-22.4)	22.3 (19.8 – 22.7)	20.6 (20.0-21.9)	21.1 (20.0-22.6)
	OB	31.0 (30.3 – 32.6) ###	33.7 (32.2-36.0)*###	31.1 (30.2 – 32.2)&###	32.5 (30.7-33.9) ###
W (cm)	NW	72.8 ± 6.2	73.7 ± 6.4	71.2 ± 5.9	76.9 ± 5.8
	OB	93.8 ± 8.7###	100.2 ± 6.3###	98.4 ± 6.3###	101.8 ± 7.7*, ###
BF (%)	NW	26.7 ± 3.1	29.0 ± 3.8	28.1 ± 4.3	31.8 ± 2.5***
	OB	38.0 ± 2.0###	38.8 ± 2.2###	38.7 ± 2.2###	40.5 ± 1.6**,###
FFM (kg)	NW	38.6 ± 3.6	38.7 ± 4.4	34.6 ± 2.9**	34.6 ± 2.9**
	OB	49.1 ± 3.9###	50.7 ± 4.3###	45.4 ± 3.4*&###	45.5 ± 3.0*& ###
RMR_{AB} (kcal/day)	NW	1334.6 ± 199.8	1295.6 ± 211.8	1225.5 ± 185.0	1145.9 ± 129.4*
	OB	1442.2 ± 295.0	1637.7 ± 347.9##	1417.0 ± 272.1#	1517.8 ± 174.8###
RMR_{BM} (kcal/kg BM/day)	NW	25.3 ± 2.8	23.9 ± 4.3	25.6 ± 3.7	22.6 ± 2.9
	OB	18.2 ± 3.1###	20.3 ± 5.8	19.3 ± 4.1###	19.9 ± 1.8##
RMR_{FFM} (kcal/kg FFM/day)	NW	34.6 ± 4.2	33.8 ± 6.3	35.5 ± 4.3	33.2 ± 4.0
	OB	29.3 ± 4.9##	32.2 ± 5.8	31.4 ± 6.2#	33.4 ± 3.4
RFO_{AB} (g/min)	NW	0.06 ± 0.02	0.05 ± 0.02	0.05 ± 0.02	0.04 ± 0.02
	OB	0.06 ± 0.03	0.08 ± 0.02##	0.06 ± 0.02	0.06 ± 0.05
RFO_{BM} (g/kg BM/min)	NW	0.0011 ± 0.0003	0.0010 ± 0.0005	0.0010 ± 0.0005	0.0007 ± 0.0004
	OB	0.0004 ± 0.0004##	0.0009 ± 0.0003	0.0008 ± 0.0004	0.0007 ± 0.0006
RFO_{FFM} (g/kg FFM/min)	NW	0.0016 ± 0.0005	0.0014 ± 0.0007	0.0013 ± 0.0007	0.0011 ± 0.0005
	OB	0.0013 ± 0.0006	0.0015 ± 0.0004	0.0013 ± 0.0006	0.0012 ± 0.0010
$\dot{V}O_{2max}$ (mL/kg BM/min)	NW	28.6 ± 4.8	26.9 ± 7.3	21.8 ± 5.0	22.3 ± 4.1
	OB	21.3 ± 4.4###	22.7 ± 5.4	20.1 ± 4.9###	19.8 ± 3.3
$\dot{V}O_{2max}$ (mL/kg FFM/min)	NW	38.4 ± 5.6	37.7 ± 10.7	38.6 ± 8.4	33.7 ± 7.9
	OB	33.2 ± 7.1#	37.1 ± 8.4	32.1 ± 8.4#	33.3 ± 5.9
Plasma vitamin C (µmol/L)	NW	6.3 ± 4.1	43.6 ± 11.7	33.5 ± 14.8	39.9 ± 20.5
	OB	9.1 ± 3.3#	67.0 ± 20.6###	57.2 ± 18.6###	61.6 ± 22.7##
Plasma MDA (µmol/mL)	NW	5.4 (3.2 - 8.6)	5.5 (3.9 - 6.3)	3.8 (3.0 - 6.3)	9.4 (5.7 - 10.9)
	OB	9.0 (6.8 - 10.8) #	7.8 (6.4 - 9.1) ##	6.9 (3.7 - 8.7)	8.4 (6.2 - 11.3)
Serum hsCRP (mg/L)	NW	0.3 (0.2 – 0.7)	0.5 (0.3 – 1.2)	0.3 (0.1 – 0.8)	0.8 (0.3 – 1.5)
	OB	4.0 (2.6 – 6.1) ###	2.5 (1.6 – 4.7) ###	2.3 (1.0 – 3.4) ##	1.9 (1.0 – 4.0) #

Data are expressed as mean ± SD and median (interquartile range) based on normal distribution. Independent t-test or Mann-Whitney U test was used for the normally and not normally distributed variables. The data were analyzed by analysis of variance (ANOVA) after test with Bonferroni correction to reveal the significance within and between groups for normal distribution data. The data with not normal distribution were analyzed by using Kruskal Wallis test. * $p < 0.05$, ** $p < 0.01$, *** $p < 0.001$ significantly different within groups compared with the group of 20-29year-old, & $p < 0.05$, && $p < 0.01$, &&& $p < 0.001$ significantly different within groups compared with the group of 30-39-year-old, \$ $p < 0.05$, \$\$\$ $p < 0.001$ significantly different within groups compared with the group of 40-49-year-old, # $p < 0.05$, ## $p < 0.01$, ### $p < 0.001$ significantly different between groups.

Abbreviation: RTL, blood leukocyte relative telomere length; NW, normal weight; OB, obesity; BM, body mass; BMI, body mass index; W, waist circumference; BF, body fat; FFM, fat-free mass; RMR_{AB}; absolute resting metabolic rate, RMR_{BM}; resting metabolic rate adjusted for body mass, RMR_{FFM}; resting metabolic rate adjusted for FFM, RFO_{AB}; absolute resting fat oxidation rate, RFO_{BM}; resting fat oxidation rate adjusted for body mass, RFO_{FFM}; resting fat oxidation rate adjusted for fat-free mass, $\dot{V}O_{2max}$ (mL/kg BM/min); maximum oxygen consumption adjusted for body mass, $\dot{V}O_{2max}$ (mL/kg FFM/min); maximum oxygen consumption adjusted for fat-free mass, MDA; malondialdehyde, hsCRP; high-sensitivity C-reactive protein.

Supplementary Table S3 Associations between RTL and body composition, metabolism, aerobic capacity, oxidative stress, and inflammation; age subgroup.

	**20-29-year-old (*n* = 34)**	**30-39-year-old (*n* = 32)**	**40-49-year-old (*n* = 34)**	**50-59-year-old (*n* = 34)**
BMI (kg/m^2)	-0.042 (-0.058; -0.026) ***	-0.028 (-0.048; -0.009) **	-0.000 (-0.017; 0.017)	-0.015 (-0.031; 0.001)
W (cm)	-0.017 (-0.024; -0.009) ***	-0.013 (-0.022; -0.004) **	-0.000 (-0.007; 0.006)	-0.0.011; 0.002)
BF (%)	-0.040 (-0.053; -0.026) ***	-0.025 (-0.046; -0.005) *	0.001 (-0.015; 0.016)	-0.015 (-0.034; 0.005)
FFM (kg)	-0.030 (-0.045; -0.015) ***	-0.019 (-0.029; 0.001)	-0.001 (-0.016; 0.014)	-0.019 (-0.034; -0.003) *
RMR_{AB} (kcal/day)	-0.000 (-0.001; -0.000) *	-0.000 (-0.001; -0.000) *	0.000 (-0.000; 0.000)	-0.000 (-0.001; 0.000)
RMR_{BM} (kcal/kg BM/day)	0.031 (0.008; 0.055) **	-0.005 (-0.028; 0.017)	0.002 (-0.016; 0.019)	0.013 (-0.022; 0.047)
RMR_{FFM} (kcal/kg FFM/day)	0.010 (-0.013; 0.032)	-0.012 (-0.032; 0.007)	0.002 (-0.012; 0.017)	-0.001 (-0.024; 0.022)
RFO_{AB} (g/min)	-2.831 (-7.319; 1.657)	-5.060 (-9.936; -0.184) *	0.836 (-2.306; 3.978)	-0.159 (-2.359; 2.042)
RFO_{BM} (g/kg BM/min)	139.6 (-168.4; 447.7)	-193.4 (-511.7; 124.9)	56.0 (-116.3; 228.3)	8.246 (-155.8; 172.3)
RFO_{FFM} (g/kg FFM/min)	-18.0 (-243.0; 207.0)	-168.2 (-377.5; 41.2)	42.5 (-79.8; 164.9)	-1.60 (-101.22; 98.02)
$\dot{V}O_{2max}$ (mL/kg BM/min)	0.011 (-0.010; 0.031)	0.011 (-0.008; 0.029)	-0.002 (-0.017; 0.013)	0.010 (-0.011; 0.031)
$\dot{V}O_{2max}$ (mL/kg FFM/min)	-0.002 (-0.019; 0.016)	0.004 (-0.009; 0.018)	0.002 (-0.008; 0.012)	0.007 (-0.005; 0.018)
Plasma MDA (µmol/mL)	-0.018 (-0.048; 0.012)	-0.017 (-0.057; 0.022)	0.001 (-0.014; 0.017)	0.000 (-0.018; 0.019)
Plasma vitamin C (µmol/L)	-0.001 (-0.007; 0.004)	-0.005 (-0.011; 0.001)	-0.001 (-0.005; 0.004)	0.003 (-0.001; 0.006)
Serum hsCRP (mg/L)	-0.064 (-0.101; -0.027) **	-0.027 (-0.086; 0.031)	-0.003 (-0.029; 0.023)	-0.025 (-0.071; 0.021)

Data indicates beta-coefficient (95% CI) for the continuous variable in a GLM adjusted for EE, EI, and EL (* $p < 0.05$, ** $p < 0.01$, *** $p < 0.001$).

Abbreviation: RTL, blood leukocyte relative telomere length; BM, body mass; BMI, body mass index; W, waist circumference; BF, body fat; FFM, fat-free mass; RMR_{AB}; absolute resting metabolic rate, RMR_{BM}; resting metabolic rate adjusted for body mass, RMR_{FFM}; resting metabolic rate adjusted for FFM, RFO_{AB}; absolute resting fat oxidation rate, RFO_{BM}; resting fat oxidation rate adjusted for body mass, RFO_{FFM}; resting fat oxidation rate adjusted for FFM, $\dot{V}O_{2max}$ (mL/kg BM/min); maximum oxygen consumption adjusted for body mass, $\dot{V}O_{2max}$ (mL/kg FFM/min); maximum oxygen consumption adjusted for fat-free mass, MDA; malondialdehyde, hsCRP; high-sensitivity C-reactive protein.

Supplementary Table S4. Associations between RTL and body composition, metabolism, aerobic capacity, oxidative stress, and inflammation; NW group.

	Model	20-29-year-old (*n* = 17)	30-39-year-old (*n* = 17)	40-49-year-old (*n* = 17)	50-59-year-old (*n* = 17)
BMI (kg/m²)	Model1	-0.107 (-0.208; -0007) *	0.009 (-0.152; 0.170)	0.040 (-0.097; 0.178)	0.016 (-0.065; 0.097)
W (cm)	Model1	-0.005 (-0.026; 0.016)	0.001 (-0.044; 0.045)	0.004 (-0.027; 0.035)	0.017 (-0.003; 0.037)
	Model3	-0.004 (-0.024; 0.016)	0.002 (-0.031; 0.036)	-0.004 (-0.029; 0.021)	0.016 (0.002; 0.030) *
BF (%)	Model1	-0.041 (-0.077; -0.005) *	0.012 (-0.053; 0.077)	0.011 (-0.034; 0.055)	0.002 (-0.051; 0.055)
FFM (kg)	Model1	-0.001 (-0.041; 0.038)	0.004 (-0.066; 0.075)	0.010 (-0.063; 0.083)	0.003 (-0.036; 0.042)
RMR_{AB} (kcal/day)	Model1	-0.000 (-0.001; 0.000)	-0.001 (-0.002; 0.000)	-0.000 (-0.001; 0.001)	0.000 (-0.001; 0.001)
	Model2	-0.000 (-0.001; 0.001)	-0.001 (-0.002; 0.000)	-0.002 (-0.004; 0.000)	0.000 (-0.001; 0.001)
RMR_{BM} (kcal/kg BM/day)	Model1	-0.005 (-0.047; 0.037)	-0.037 (-0.083; 0.009)	-0.018 (-0.065; 0.029)	0.007 (-0.030; 0.044)
	Model2	-0.019 (-0.068; 0.030)	-0.056 (-0.124; 0.012)	-0.083 (-0.187; 0.020)	0.008 (-0.046; 0.061)
RMR_{FFM} (kcal/kg FFM/day)	Model1	-0.008 (-0.040; 0.025)	-0.025 (-0.057; 0.007)	-0.014 (-0.054; 0.026)	0.006 (-0.021; 0.034)
	Model4	0.001 (-0.029; 0.031)	-0.033 (-0.063; -0.003) *	-0.004 (-0.037; 0.030)	0.005 (-0.015; 0.026)
RFO_{AB} (g/min)	Model1	-1.848 (-7.378; 3.683)	-4.119 (-14.585; 6.348)	1.269 (-5.385; 7.922)	-1.923 (-7.987; 4.143)
RFO_{BM} (g/kg BM/min)	Model1	-112.9; (-476.4; 250.6)	-217.5 (-670.5; 235.6)	51.7 (-247.0; 350.5)	-113.7 (-413.4; 186.0)
RFO_{FFM} (g/kg FFM/min)	Model1	-95.8 (-344.1; 152.6)	-145.2 (-476.1; 185.7)	47.9 (-176.9; 272.6)	-86.9 (-299.0; 125.1)
$\dot{V}O_{2max}$ (mL/kg BM/min)	Model1	-0.011 (-0.034; 0.013)	0.001 (-0.333; 0.359)	-0.011 (-0.046; 0.024)	-0.012 (-0.037; 0.012)
	Model2	-0.012 (-0.041; 0.016)	0.022 (-0.038; 0.082)	-0.016 (-0.065; 0.033)	-0.012 (-0.042; 0.018)
$\dot{V}O_{2max}$ (mL/kg FFM/min)	Model1	-0.010 (-0.031; 0.012)	0.004 (-0.024; 0.032)	-0.002 (-0.022; 0.019)	-0.003 (-0.018; 0.012)
	Model2	-0.010 (-0.037; 0.017)	0.013 (-0.036; 0.052)	-0.011 (-0.046; 0.023)	-0.002 (-0.021; 0.016)
	Model3	-0.005 (-0.025; 0.014)	0.020 (0.000; 0.040) *	0.003 (-0.015; 0.021)	-0.001 (-0.013; 0.011)
Plasma MDA (μmol/mL)	Model1	-0.010 (-0.039; 0.018)	-0.026 (-0.087; 0.035)	0.051 (-0.020; 0.122)	0.006 (-0.015; 0.028)
Plasma vitamin C (μmol/L)	Model1	0.004 (-0.003; 0.011)	0.000 (-0.018; 0.019)	0.003 (-0.012; 0.019)	0.004 (-0.001; 0.008)
	Model3	0.003 (-0.003; 0.010)	-0.001 (-0.019; 0.018)	-0.003 (-0.013; 0.007)	0.004 (0.000; 0.008) *
Serum hsCRP (mg/L)	Model1	-0.122 (-0.407; 0.163)	0.026 (-0.326; 0.377)	-0.015 (-0.074; 0.044)	-0.018 (-0.076; 0.041)

Data indicates beta-coefficient (95% CI) for the continuous variable in a GLM in NW participants by subgroup of ages (* $p < 0.05$). Model 1 = adjusted for age, energy expenditure, energy intake, and education level; Model 2 = model1 add BMI, W, body fat (%) and FFM; Model 3 = FBG; Model 4 = HOMA-IR.

Abbreviation: RTL, blood leukocyte relative telomere length; NW, normal weight; BM, body mass; BMI, body mass index; W, waist circumference; BF, body fat; FFM, fat-free mass; RMR_{AB}; absolute resting metabolic rate, RMR_{BM}; resting metabolic rate adjusted for body mass, RMR_{FFM}; resting metabolic rate adjusted for fat-free mass, RFO_{AB}; absolute resting fat oxidation rate, RFO_{BM}; resting fat oxidation rate adjusted for body mass, RFO_{FFM}; resting fat oxidation rate adjusted for fat-free mass, $\dot{V}O_{2max}$ (mL/kg BM/min); maximum oxygen consumption adjusted for body mass, $\dot{V}O_{2max}$ (mL/kg FFM/min); maximum oxygen consumption adjusted for fat-free mass, MDA; malondialdehyde, hsCRP; high-sensitivity C-reactive protein.

Supplementary Table S5. Associations between RTL and body composition, metabolism, aerobic capacity, oxidative stress, and inflammation; OB group.

	Model	20-29-year-old (*n* = 17)	30-39-year-old (*n* = 15)	40-49-year-old (*n* = 17)	50-59-year-old (*n* = 17)
BMI (kg/m^2)	Model1	0.035 (-0.045; 0.116)	-0.012 (-0.042; 0.019)	0.005 (-0.052; 0.062)	-0.017 (-0.083; 0.049)
W circumference (cm)	Model1	0.004 (-0.019; 0.027)	-0.012 (-0.028; 0.004)	-0.005 (-0.018; 0.009)	0.001 (-0.018; 0.019)
	Model6	0.00 (-0.014; 0.018)	-0.003 (-0.017; 0.011)	-0.011 (-0.022; -0.000) *	-0.006 (-0.025; 0.013)
BF (%)	Model1	0.017 (-0.081; 0.114)	-0.029 (-0.067; 0.010)	0.006 (-0.041; 0.043)	-0.007 (-0.106; 0.091)
FFM (kg)	Model1	0.003 (-0.038; 0.045)	0.004 (-0.019; 0.027)	-0.010 (-0.038; 0.017)	-0.033 (-0.074; 0.007)
	Model4	-0.006 (-0.042; 0.031)	0.007 (-0.013; 0.026)	-0.012 (-0.033; 0.008)	-0.042 (-0.076; -0.007)*
RMR_{AB} (kcal/day)	Model1	-0.000 (-0.001; 0.000)	0.000 (-0.000; 0.000)	0.000 (0.000; 0.001)	-0.000 (-0.001; 0.000)
	Model2	-0.001 (-0.002; 0.000)	0.000 (-0.000; 0.000)	0.000 (0.000; 0.001) **	-0.000 (-0.002; 0.001)
RMR_{BM} (kcal/kg BM/day)	Model1	-0.043 (-0.087; 0.001)	-0.003 (-0.020; 0.014)	0.024 (0.004; 0.044) *	-0.009 (-0.085; 0.067)
	Model2	-0.060 (-0.128; 0.009)	-0.003 (-0.022; 0.019)	0.036 (0.018; 0.055) ***	-0.035 (-0.144; 0.075)
RMR_{FFM} (kcal/kg FFM/day)	Model1	-0.030 (-0.058; -0.002) *	-0.000 (-0.016; 0.016)	0.017 (0.004; 0.029) *	-0.005 (-0.046; 0.036)
	Model2	-0.036 (-0.078; 0.005)	0.003 (-0.012; 0.017)	0.022 (0.010; 0.033) ***	-0.020 (-0.084; 0.044)
	Model3	-0.023 (-0.054; 0.007)	-0.001 (-0.016; 0.014)	0.011 (0.001; 0.022) *	0.004 (-0.031; 0.039)
RFO_{AB} (g/min)	Model1	-3.072 (-7.755; 1.611)	-0.811 (-5.541; 3.918)	2.385 (-1.679; 6.449)	-0.050 (-3.132; 3.031)
RFO_{BM} (g/kg BM/min)	Model1	-256.9 (-624.8; 111.0)	-51.2 (-434.4; 332.0)	161.8 (-94.7; 418.3)	14.5 (-224.8; 253.8)
RFO_{FFM} (g/kg FFM/min)	Model1	-166.2 (-404.6; 72.2)	-48.1 (-262.7; 166.5)	108.6 (-60.4; 277.8)	4.6 (-138.4; 147.6)
$\dot{V}O_{2max}$ (mL/kg BM/min)	Model1	-0.043 (-0.066; -0.021) ***	0.001 (-0.017; 0.018)	0.013 (-0.010; 0.036)	0.018 (-0.036; 0.072)
	Model2	-0.052 (-0.081; -0.023) ***	0.004 (-0.019; 0.026)	0.026 (-0.009; 0.062)	0.029 (-0.034; 0.091)
$\dot{V}O_{2max}$ (mL/kg FFM/min)	Model1	-0.029 (-0.043; -0.014) ***	-0.001 (-0.012; 0.010)	0.015 (0.005; 0.027) **	0.016 (-0.012; 0.043)
	Model2	-0.028 (-0.046; -0.011) **	0.002 (-0.013; 0.017)	0.021 (0.009; 0.033) **	0.014 (-0.017; 0.045)
	Model5	-0.022 (-0.036; -0.008) **	-0.002 (-0.012; 0.008)	0.006 (-0.002; 0.015)	0.019 (0.001; 0.038) *
Plasma MDA (μmol/mL)	Model1	0.016 (-0.038; 0.070)	-0.002 (-0.057; 0.052)	-0.006 (-0.019; 0.006)	-0.015 (-0.046; 0.016)
Plasma vitamin C (μmol/L)	Model1	0.004 (-0.004; 0.011)	-0.002 (-0.006; 0.003)	-0.001 (-0.006; 0.004)	0.005 (-0.001; 0.010)
	Model3	0.002 (-0.003; 0.008)	-0.001 (-0.005; 0.003)	-0.000 (-0.005; 0.005)	0.006 (0.001; 0.010) *
Serum hsCRP (mg/L)	Model1	-0.008 (-0.059; 0.043)	0.017 (-0.018; 0.051)	0.012 (-0.021; 0.045)	-0.071 (-0.175; 0.032)

Data indicates beta-coefficient (95% CI) for the continuous variable in a GLM in OB participants by subgroup of ages (* $p < 0.05$, ** $p < 0.01$, *** $p < 0.001$); Model 1 = adjusted for age, energy expenditure, energy intake, and education level; Model 2 = model1 add BMI, W circumference, body fat (%) and FFM; Model 3 = FBG; Model 4 = HOMA-IR; Model 5 = HbA1c; Model 6 = FBG+HOMA-IR.

Abbreviation: RTL, blood leukocyte relative telomere length; OB, obesity; BM, body mass; BMI, body mass index; W, waist circumference; BF, body fat; FFM, fat-free mass; RMR_{AB}; absolute resting metabolic rate, RMR_{BM}; resting metabolic rate adjusted for body mass, RMR_{FFM}; resting metabolic rate adjusted for FFM, RFO_{AB}; absolute resting fat oxidation rate, RFO_{BM}; resting fat oxidation rate adjusted for body mass, RFO_{FFM}; resting fat oxidation rate adjusted for fat-free mass, $\dot{V}O_{2max}$ (mL/kg BM/min); maximum oxygen consumption adjusted for body mass, $\dot{V}O_{2max}$ (mL/kg FFM/min); maximum oxygen consumption adjusted for fat-free mass, MDA; malondialdehyde, hsCRP; high-sensitivity C-reactive protein.
